# Supplementary material for: Toxicological Assessment of 2-Hydroxychalcone-Mediated Photodynamic Therapy: Comparative In Vitro and In Vivo Approaches
Source: Pharmaceutics. 2024 Nov 26;16(12):1523. doi: 10.3390/pharmaceutics16121523 (PMC11728496; doi:10.3390/pharmaceutics16121523)
Supplement: Supplementary file 1 [file pharmaceutics-16-01523-s001.zip › pharmaceutics-3274555-supplementary.pdf]

pharmaceutics MDPI

SUPPLEMENTARY MATERIAL

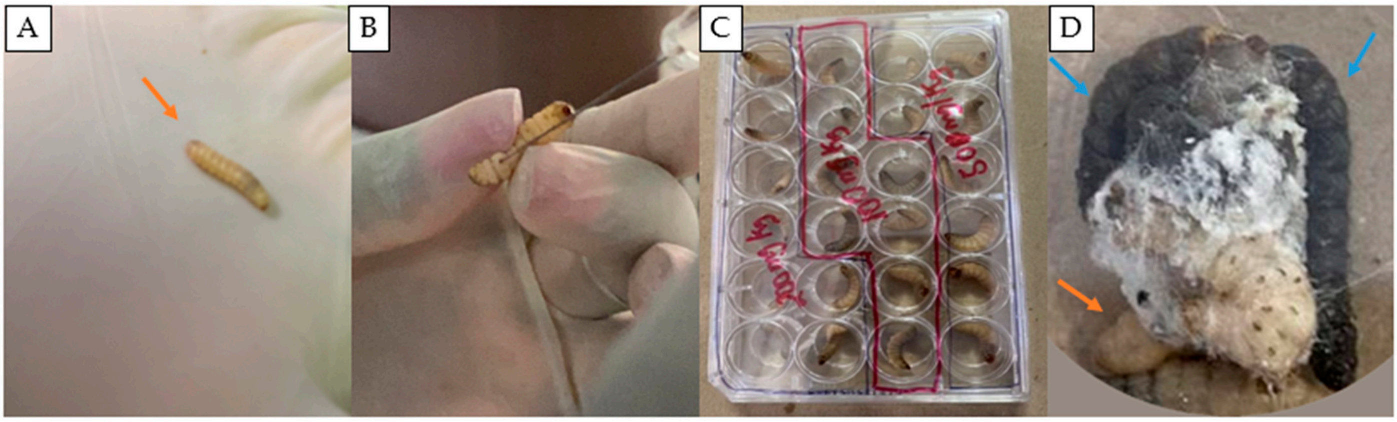

Figure S1. Illustrative images of *Galleria mellonella* larvae during the experimental process. **(A)** Live larva (orange arrow), weighing 0.2 to 0.3 g, without dark spots. **(B)** Experimental procedure: ten microliters of the solutions were injected into the last pair of prolegs (always the right proleg) using a Hamilton syringe. **(C)** After injection of 2-hydroxychalcone, the larvae were placed in 24-well plates to be irradiated with blue light. **(D)** Seventh day of the experiment, presence of dead and melanized larvae (blue arrows) and presence of live larvae (orange arrow).
